# Uncovering the Phytochemical Profile, Antioxidant Potency, Anti-Inflammatory Effects, and Thrombolytic Activity in *Dendrobium lindleyi* Steud.

**DOI:** 10.1155/2023/9999640

**Published:** 2023-09-27

**Authors:** Minhajur Rahman, Roxy Begum, Abu Taleb Surag, Md. Shakhuat Hossain Tusher, Mohammed Kamrul Huda

**Affiliations:** ^1^Department of Botany, Faculty of Biological Sciences, University of Chittagong, Chattogram, Bangladesh; ^2^Ecology and Phytochemistry Lab, Department of Botany, Faculty of Biological Sciences, University of Chittagong, Chattogram, Bangladesh; ^3^In Silico Drug Design Lab, Department of Botany, Faculty of Biological Sciences, University of Chittagong, Chattogram, Bangladesh; ^4^Department of Pharmacy, Faculty of Science & Engineering, Southern University, Chattogram, Bangladesh

## Abstract

*Background. Dendrobium* genus has been used in traditional medicine to treat various illnesses. The study aims at examining the phytochemical, antioxidant, anti-inflammatory, and thrombolytic properties of the leaf, stem, and root of *Dendrobium lindleyi *Steud, and the relationship between phytochemicals and bioactivities is determined. *Results*. The qualitative screening found a variety of bioactive compounds, including alkaloids, coumarins, cardiac glycosides, glycosides, flavonoids, proteins, phenols, quinines, resins, steroids, saponins, tannins, and terpenoids, in varying amounts. The quantitative screening showed the highest concentration of alkaloids in the leaves (172.15 ± 1.22 mg/g), phenols in the root (203.55 ± 0.75 mg/g), flavonoids in the root (24.35 ± 0.42 mg/g), tannins in the leaves (105.06 ± 0.55 mg/g), and proteins in the root (194.12 ± 0.65 *µ*g/ml). The root extract showed the highest antioxidant activity (IC_50_ = 58.24 *µ*g/mL), the stem extract had the most increased thrombolytic activity (IC_50_ = 242.74 *µ*g/mL), and the leaf extract had the most potent anti-inflammatory activity (IC_50_ = 61.79 *µ*g/mL). Statistical analysis revealed a significant positive relationship (*p* = 0.05) between alkaloids (*r* = 0.96) and tannins (*r* = 0.9) with antioxidant, anti-inflammatory, and thrombolytic properties. *Conclusion*. The bioactivities of *D. lindleyi*, including antioxidant (root), thrombolytic (stem), and anti-inflammatory (leaf) activities, are linked to the phytochemicals detected in the screening.

## 1. Background

The traditional medical systems of Ayurvedic, Siddha, and Unani have documented the use of orchids as medicine, as recorded by Charaka, Sushruta, and Vagbhata. With the growing demand for natural remedies, there is an increased interest in the medicinal use of orchids in traditional medicine [1]. The use of *Dendrobium* species as medicine has a long history, as it was mentioned in the Chinese pharmacopeia “The Sang Nung Pen Tsao Ching” as early as 200 B.C. as a tonic, astringent, analgesic, and anti-inflammatory substance [2]. Emperor Shen-Nung also described the medicinal benefits of *Dendrobium* in his “Materia medica.” Modern research has confirmed the anti-inflammatory, antifungal, antibacterial, and other pharmacological properties of *Dendrobium* species [3, 4]. Bangladesh has approximately 194 species of orchids, including 91 medicinal orchids [5–9]. *Dendrobium lindleyi* Steud. It is one of the orchid species found in Bangladesh. Although its traditional use or therapeutic benefits are unknown, fresh and dried stems of *D. lindleyi* are used in traditional Chinese medicine to treat dehydration, fever, and eye health [10]. Previous research has shown promising biological activity in the floral section of the plant [11]; however, the biological activity of the leaves, stem, and root of the plant still needs to be explored. Therefore, a comprehensive investigation of the phytochemical and pharmacological aspects of *D. lindleyi* is being conducted. The study aims at validating its traditional uses and creating a foundation for further research towards developing new drugs from this species.

## 2. Methods

### 2.1. Reagents and Chemicals

Dragendroff's reagent, Hager's reagent, Wagner's reagent, Tannic acid reagent, Mayer's reagent, Folin–Ciocalteau reagent, 10% NaOH, glacial acetic acid, ferric chloride, conc. sulphuric acid, ethyl acetate, dilute ammonia solution, methanol, 50% HCL, Fehling's solution, acetic acid anhydride, chloroform, ethanol, potassium hydroxide pellets, ammonium hydroxide, sodium nitrite, aluminum chloride, sodium hydroxide, quercetin, sodium carbonate, gallic acid, tannic acid, NaOH solutions, NaK tartrate, CuSO_4,_ bovine serum albumin, 1,1-diphenyl-2-picrylhydrazyl, ascorbic acid, egg albumin, iso-saline, acetyl salicylic acid, streptokinase vial (15,00,000 I.U.), and blood sample.

### 2.2. Plant Collection and Identification

Plants were collected from Bandarban (Keokradong hill), Chittagong, in the southeast region of Bangladesh, in April 2019. The area lies within latitude 22°11′43″ North and longitude 92°13′10″ East and runs along the border of Bangladesh. The plant was identified in the Chittagong University Herbarium (CTGUH), Department of Botany, University of Chittagong, where a voucher specimen (Accession no: KB_05/2019) was deposited for future reference.

### 2.3. Plant Extraction

Fresh leaves, stems, and roots were harvested and thoroughly washed with water, room dried, and sun-dried. Then, the samples were dried in an oven at 60° for 72 hours. It was then ground into a coarse powder using a grinding machine and stored in an air-tight container for further investigation. 25 gm of sample from each plant part were taken for further analysis. 150 ml of methanol was added to each sample in a conical flask. It was then shaken well for 30 minutes, kept overnight, shaken again, and sonicated for 10 minutes. It was then filtered using the Whatman No. 1 filter paper. The process was repeated thrice with methanol; then, the extract was evaporated below 51° and dried. The dried sample was kept as a crude sample for each part.

### 2.4. Phytochemical Investigation

#### 2.4.1. Qualitative

Qualitative tests were carried out on the crude and powdered extracts of leaves, stems, and roots of *D. lindleyi* using standard procedures [12–14] to identify alkaloids, coumarins, cardiac glycosides, glycosides, flavonoids, proteins, phenols, quinines, resins, steroids, saponins, tannins, and terpenoids. The presence of relative phytochemicals in the extract of test samples was expressed by the “+” sign ranging in the order of “+,” “++,” and “+++” signifying its presence in degrees (“+” the lowest, “++” the moderate, and' “+++” the highest quantity). The “-” sign denoted the absence of the phytochemicals.

#### 2.4.2. Quantitative

Quantitative phytochemical tests were performed using the following standard procedures.

*(1) Total Alkaloid Determination*. For determining the total alkaloid content, fresh samples of leaves, roots, and stems (5 g each) were placed in 250 mL beakers and a mixture of 200 mL 20% acetic acid in ethanol was added. After 4 hours, the mixture was filtered and the extract was concentrated in a water bath to a quarter of its original volume. Concentrated ammonium hydroxide was gradually added until precipitation was complete. After settling, the precipitate was washed, filtered, dried, and weighed with the weight expressed as mg/g extract [15].

*(2) Total Flavonoid Determination*. For total flavonoid estimation, standard solutions of quercetin were prepared and plant extracts were treated similarly. After incubation and spectral measurement at 510 nm, the flavonoid content was expressed in mg quercetin equivalents (QE)/g of the dried plant extract [16, 17].

*(3) Total Phenol Determination*. Methanol extracts (1 mg/ml) were analyzed to determine the total phenol concentration. A mixture of methanol extract, 10% Folin–Ciocalteu's reagent, and 7.5% sodium carbonate solution was prepared and incubated. Absorbance was measured at 760 nm using a UV spectrophotometer. The total phenol concentration was determined from a calibration curve of gallic acid equivalents (GAE) and expressed as mg GAE/g of the extract [18, 19].

*(4) Total Tannin Determination*. For total tannin determination, extracts (1 mg/ml) were mixed with water, Folin–Ciocalteu reagent, sodium carbonate solution, and distilled water. After incubation, absorbance at 700 nm was measured using a UV spectrophotometer. Tannin content was expressed in mg of tannic acid equivalents/g of the dried sample [20].

*(5) Total Protein Determination*. A phenolic complex with the maximum absorption at 660 nm was formed for total protein determination. Bovine serum albumin (BSA) was used as a standard protein. Samples were mixed with reagents and incubated. Then, absorbance was measured at 660 nm using a UV spectrophotometer. The protein content was expressed in mg/g of dried plant extract [21].

### 2.5. In Vitro Analysis

#### 2.5.1. Antioxidant Activity

The antioxidant activities of the methanolic crude extracts of the leaves, stems, and roots of *D. lindleyi* and the standard antioxidant ascorbic acid were assessed based on the free radical scavenging effect of the stable 2, 2-diphenyl-1-picrylhydrazyl (DPPH, MWt. 394.32) free radical activity with slight modification [22]. Preparation of reagents of crude extract of the leaves, stems, and roots of *D. lindleyi* was conducted to prepare a range of concentrations (50, 100, 150, 200, and 250 *µ*g/mL) in methanol. Ascorbic acid (*Positive control*) with similar concentrations was also prepared in methanol. 0.004% DPPH solution was prepared in methanol. The absorbance was measured at 517 nm using a UV-visible spectrophotometer. The experiment was performed thrice. The scavenging activity against DPPH was calculated using the following equation:

(1) $$\begin{array}{c} \text{scavenging activity }\left(\%\right)=\left(\frac{A-B}{A}\right)\times 100\text{,} \end{array}$$

where *A* = Absorbance of DPPH solution (*negative control*) and *B* = Absorbance of DPPH solution (extracts/ascorbic acid).

#### 2.5.2. Anti-Inflammatory Activity

The anti-inflammatory activities of the methanolic crude extracts of the leaf, stem, and root of *D. lindleyi* and the standard anti-inflammatory agent acetyl salicylic acid were assessed based on inhibition of the albumin denaturation technique of the stable egg albumin denaturation inhibition activity with slight modification [23, 24]. Preparation of reagents of crude extracts of the leaf, stem, and root of *D. lindleyi* was carried out to prepare a range of concentrations (50, 100, 150, 200,250, and 300 *µ*g/mL) in methanol and 1% aqueous solution of egg albumin, and the pH values (5.6 ± 0.2) of all mixtures were adjusted by 1N HCl. Acetylsalicylic acid with different concentrations (50, 100, 150, 200, 250, and 300 *µ*g/mL) was also prepared. The absorbance was measured at 660 nm using a UV-visible spectrophotometer. The experiment was performed thrice. The anti-inflammatory activity was calculated by using the following equation:

(2) $$\begin{array}{c} \%\text{ of inhibition}=\left(\frac{A-B}{A}\right)\times 100\text{,} \end{array}$$

where *A* = absorbance of egg albumin solution + methanol (*negative control*) and *B* = absorbance of egg albumin solution + extract/standard (*positive control*).

#### 2.5.3. Thrombolytic Activity

A clot lysis experiment was carried out to check the thrombolytic properties of the plant extract. In this method [25], venous blood drawn from healthy volunteers are transferred in different preweighed sterile Eppendorf tubes (500 *μ*L/tube) and incubated at 37°C for 45 minutes. After clot formation, the serum is completely removed (aspirated out without disturbing the clot formed). Each tube having a clot is again weighed to determine the clot weight. Each Eppendorf tube containing the clot is labelled correctly, and 100 *μ*L of plant extract is added to the tubes. All the tubes are then incubated at 37°C for 90 minutes and observed for clot lysis. After incubation, the fluid obtained is removed and tubes are again weighed to keep the difference in weight after clot disruption. Streptokinase and water are *positive* and *negative* (nonthrombolytic) controls, respectively. The experiment is repeated three times with the blood samples of different volunteers. The difference obtained in weight taken before and after clot lysis is expressed as a percentage of clot lysis as follows:

(3) $$\begin{array}{c} \text{clot weight}=\text{weight of clot}-\text{containing tube}-\text{the weight of tube alone,}\\ \%\text{ of clot lysis}=\left(\frac{\text{weight of released clot}}{\text{clot weight}}\right)\times 100\text{.} \end{array}$$

#### 2.5.4. Statistical Analysis

Finally, the study statistically examined total phytochemicals (alkaloids, phenols, tannins, flavonoids, and proteins) for their effect on bioactivity (antioxidant, anti-inflammatory, and thrombolytic). For this, regression analysis and Pearson's correlation coefficient analysis were performed by Microsoft Excel 2010.

## 3. Results

### 3.1. Phytochemical Screening

#### 3.1.1. Qualitative

The responses of the plant extracts to various reagents provide valuable insights into their chemical composition and the presence of specific alkaloid groups in different parts of the plant (Table 1). When tested with Dragendorff's reagent, the leaf extract displayed a “++” response, the stem extracted a “+++” reply, and the root extracted a “++” response. Similarly, when exposed to Hager's reagent, all parts of the plant—the leaf, stem, and root—demonstrated a “+++” response. Mayer's reagent produced distinct responses as well. The leaf extract exhibited a “+++” response, the stem extracts a “+” reply, and the root extract a “+++” response. Furthermore, against Wagner's reagent, the leaf extract showcased a “+++” response, the stem extracts a “++” response, and the root extract a “++” reply. Lastly, the tannic acid reagent elicited “++,” “+,” and “-” responses from the leaf, stem, and root extracts.

Moreover, coumarins, cardiac glycosides, flavonoids, proteins, phenols, quinines, resins, steroids, saponins, tannins, and terpenoids were detected in varied amounts during the qualitative screening (Table 2). The leaf, stem, and root reported traces of glycosides and saponins. The resin was evident in the root but just a tiny amount in the stem and none in the leaf.

#### 3.1.2. Quantitative

*(1) Total Alkaloid*. Total alkaloid contents of *D. lindleyi* leaf, root, and stem recorded were 172.15 ± 1.22, 109.47 ± 0.93, and 148.4 ± 1.00 mg/g, respectively. Leaf exhibited the highest alkaloid contents of 172.15 ± 1.22 mg/g.

*(2) Total Flavonoid*. In the present work, 13.86 ± 0.20, 1.63 ± 0.66, and 24.35 ± 0.42 mg·g^−1^ of flavonoids were found in *D. lindleyi* in the leaf, stem, and root, respectively. The highest amount of flavonoid found in the root extract was 24.35 ± 0.42 mg/g.

*(3) Total Phenol*. Total phenol concentrations of *D. lindleyi* leaf, root, and stem were 176.47 ± 1.05 mg/g, 148.22 ± 0.82 mg/g, and 203.55 ± 0.75 mg/g, respectively, in quantitative evaluations. The root has the highest phenol concentration, measuring 203.55 ± 0.75 mg/g.

*(4) Total Protein*. The highest amount of protein content detected in the root methanolic extracts was 194.12 ± 0.65 *µ*g/ml, equivalent to BSA, whereas the leaf and stem methanolic crude extracts of *D. lindleyi* contain (178.97 ± 0.41 and 131.61 ± 0.49) BSA identical µg/ml plant extract, respectively.

*(5) Total Tannin*. The total tannin contents of the leaf, stem, and root of *D. lindleyi* were found to be (105.06 ± 0.55, 53.12 ± 1.34, and 69.60 ± 1.21) mg·g^−1^ respectively. The highest amount of tannin in the leaf extract was 105.06 ± 0.55 mg/g compared to the extracts of the stem and the root.

**Figure 1:**
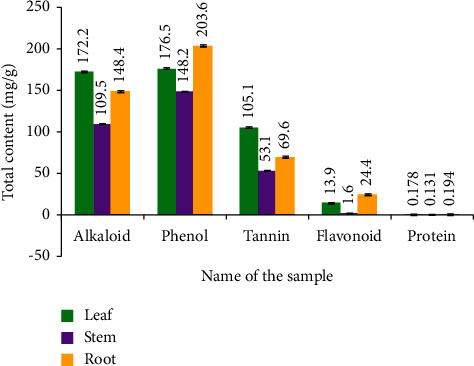
Total alkaloid, phenol, tannin, flavonoid, and protein content of *D. lindleyi*. Note: values are represented as the mean ± standard error mean (*n* = 3).

Thus, the plant revealed a maximum of 172.15 ± 1.22 mg/g alkaloids (leaf), 203.55 ± 0.75 mg/g phenols (root), 24.35 ± 0.42 mg/g flavonoids (root), 105.06 ± 0.55 mg/g tannins (leaf), and 194.12 ± 0.65 *µ*g/mL proteins (root) in quantitative screening (Figure 1).

### 3.2. In Vitro Analysis

#### 3.2.1. Antioxidant Activity

**Figure 2:**
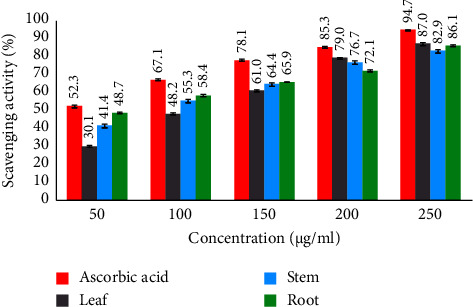
Antioxidant activity of *D. lindleyi*.

**Figure 3:**
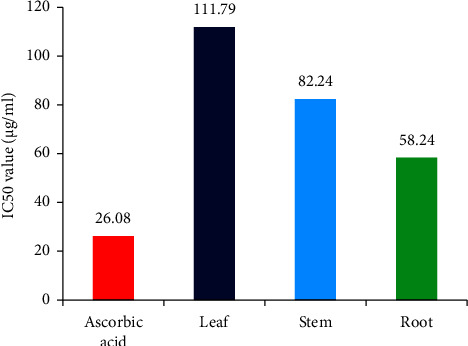
IC_50_ values (antioxidant) of standard and plant parts.

The antioxidant activity was assessed using the DPPH free radical scavenging assay. The antioxidant activity (Figure 2) of leaf, stem, and root extracts obtained IC_50_ values of 111.79, 82.24, and 58.24 *µ*g/mL, respectively. Compared to the standard (26.08 *µ*g/mL), the root extract showed the most potent antioxidant activity, the stem extract displayed intermediate potency, and the leaf extract exhibited minor antioxidant potency among the tested plant parts (Figure 3).

#### 3.2.2. Anti-Inflammatory Activity

**Figure 4:**
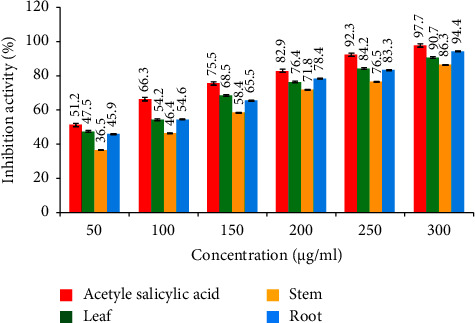
Anti-inflammatory activity of *D. lindleyi*.

**Figure 5:**
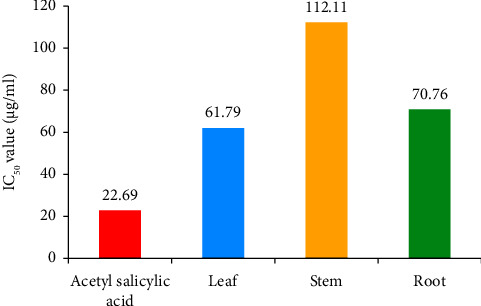
IC_50_ values (anti-inflammatory) of standard and plant parts.

The anti-inflammatory activity was evaluated through an albumin denaturation assay, which measures the ability of substances to prevent the denaturation (unfolding) of albumin, a protein. The IC_50_ values for the standard leaf, stem, and root extracts indicate the concentrations at which these substances effectively inhibit albumin denaturation by 50%. The leaf, stem, and root extracts had IC_50_ values of 61.79, 112.11, and 70.76 *µ*g/mL, respectively (Figure 4), with the leaf extract having the most potent anti-inflammatory effect when compared to the standard (22.69 *µ*g/mL). The IC_50_ values from the albumin denaturation assay measured the ability of the tested substances (standard, leaf, stem, and root extracts) to prevent the denaturation of albumin. A lower IC_50_ value indicates more potent anti-inflammatory activity. The standard had the most powerful protective effect, followed by the root extract, the leaf extract, and the stem extract (Figure 5). These findings give insights into the relative strengths of these substances in preserving the structural integrity of albumin.

#### 3.2.3. Thrombolytic Activity

**Figure 6:**
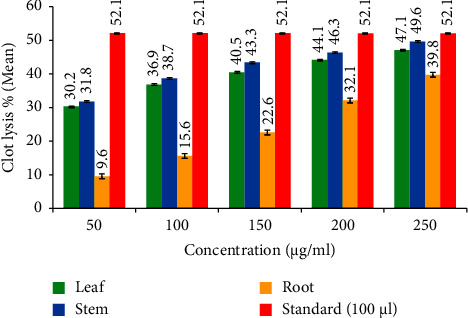
Thrombolytic activity of *D. lindleyi*.

**Figure 7:**
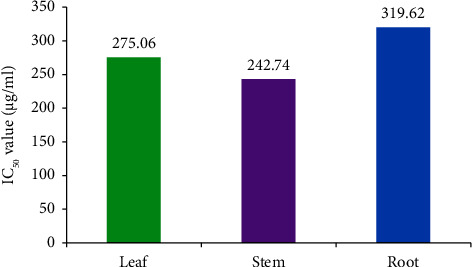
IC_50_ values (thrombolytic) of plant parts.

The thrombolytic activity was measured using the blood clotting inhibition method. The results of a thrombolytic activity assay conducted on various substances, including leaf extract, stem extract, and root extract, with IC_50_ values of 275.06, 242.74, and 319.62 *µ*g/mL, respectively, as compared to the standard (100 *µ*L) (Figure 6). The percentages of clot lysis and the IC_50_ values provide insights into their effectiveness in promoting clot dissolution. The thrombolytic activity was measured using the blood clotting inhibition method. The results suggest that the standard and the tested extracts have varying levels of thrombolytic activity. The stem extract showed the most potent thrombolytic activity, followed by the leaf extract. In contrast, the root extract exhibited the lowest thrombolytic activity (Figure 7). The IC_50_ values indicate the concentration of each extract needed to achieve a 50% reduction in clot formation, with lower values suggesting more substantial thrombolytic effects.

#### 3.2.4. Statistical Analysis

In this analysis (Table 3), correlation values (*r*) were calculated as 0.96 for alkaloids, 0.9 for tannins, 0.7 for proteins, 0.51 for phenols, and 0.54 for flavonoids which indicated a strong positive correlation for alkaloids as well as tannins, whereas they indicated moderate positive correlation for proteins, flavonoids, and phenols. Statistical analysis showed that alkaloids (*r* = 0.96) and tannin (*r* = 0.9) have a substantial positive connection with antioxidant, anti-inflammatory, and thrombolytic activities at a significance level of *p* < 0.05 significantly.

## 4. Discussion

In the phytochemical investigation, five reagents tested alkaloids. When tested with Dragendorff's reagent, the leaf extract displayed a “++” response, the stem extracted a “+++” reply, and the root extracted a “++” response. This variation in responses suggests that the stem extract likely contains a higher concentration of alkaloids with secondary amine groups than the leaf and root extracts, which showed more moderate responses. Similarly, when exposed to Hager's reagent, all parts of the plant—the leaf, stem, and root—demonstrated a “+++” response. This collective strong response strongly indicates the presence of alkaloids containing methoxy or catechol groups in substantial quantities across the entire plant. Mayer's reagent produced distinct responses as well. The leaf extract exhibited a “+++” response, the stem extracts a “+” reply, and the root extract a “+++” response. Here, the leaf and root extracts demonstrated a high response, suggesting the significant presence of alkaloids containing hydroxyl or amino groups. In contrast, the stem extract yielded a lower response to Mayer's reagent. Furthermore, against Wagner's reagent, the leaf extract showcased a “+++” response, the stem extract showcased a “++” response, and the root extract showcased a “++” reply. Notably, the leaf extract displayed the highest response, indicating the likely presence of alkaloids characterized by tertiary amines and phenolic hydroxyl groups. While the stem and root extracts showed moderate responses, they target different alkaloid characteristics. Lastly, the tannic acid reagent elicited “++,” “+,” and “-” responses from the leaf, stem, and root extracts. The leaf extract's “++” response suggests the likely presence of alkaloids that can react with tannic acid. Comparatively, the stem extract's lower “+” response suggests a lower quantity of these alkaloids, and the root extract's “-” response indicates an absence of reaction to this particular reagent. When the other phytochemical components of *D. lindleyi* were studied, a variety of bioactive compounds, including coumarins, cardiac glycosides, flavonoids, proteins, phenols, quinines, resins, steroids, saponins, tannins, and terpenoids, were discovered. The highest concentration of alkaloids was found in the leaves, while the root contained the highest levels of phenols and flavonoids. Traces of glycosides and saponins were found in the leaves, stems, and roots, and resin was only present in the root. These findings are consistent with the previous studies [26–30]. The presence of bioactive phytochemicals in *D. lindleyi* suggests that it may have a wide range of therapeutic properties [31].

The antioxidant activity was assessed using a free radical scavenging assay. An IC_50_ value of 111.79 *µ*g/mL was obtained for the leaf extract, indicating that it has comparatively lower antioxidant potency than the stem and root extracts. This means that a higher concentration of the leaf extract is required to achieve the same level of antioxidant protection as the other extracts. In contrast, the stem extract demonstrated greater antioxidant potency than the leaf extract. Its IC_50_ value was 82.24 *µ*g/mL, suggesting that it can achieve a 50% reduction in oxidative damage at a lower concentration than the leaf extract. The most robust antioxidant activity was observed in the root extract, which exhibited the lowest IC_50_ value of 58.24 *µ*g/mL among the three extracts. This signifies that the root extract possesses the highest antioxidant potency. The antioxidant effects are thought to be due to flavonoids, saponins, and phenolic substances [32, 33].

The anti-inflammatory efficacy was determined using the heat-induced albumin denaturation assay. The standard substance demonstrated an IC_50_ value of 22.69 *µ*g/mL. This means a standard substance concentration of 22.69 *µ*g/mL effectively prevents albumin denaturation by 50%. The lower the IC_50_ value, the more potent the substance is in preserving the native structure of albumin. The leaf extract had an IC_50_ value of 61.79 *µ*g/mL. At a 61.79 *µ*g/mL concentration, the leaf extract can protect albumin from denaturation by 50%. The stem extract showed an IC_50_ value of 112.11 *µ*g/mL. At a 112.11 *µ*g/mL concentration, the stem extract can inhibit albumin denaturation by 50%. The root extract displayed an IC_50_ value of 70.76 *µ*g/mL. At a 70.76 *µ*g/mL concentration, the root extract can protect albumin from denaturation by 50%. This value positions the root extract as relatively more effective in preserving the native structure of albumin than the stem extract but still less effective than the standard. The anti-inflammatory properties may be due to saponins, terpenoids, alkaloids, glycosides, and tannins [34, 35].

The thrombolytic activity was measured using the blood clotting inhibition assay. The standard exhibited a clot lysis percentage of 52.1%. This means that the standard, when used at a volume of 100 *µ*L, could cause a 52.1% dissolution of the blood clot. The standard likely represents a *positive control* with known thrombolytic activity. The leaf extract showed a range of clot lysis percentages between 30.2% and 47.1%. This suggests that the leaf extract has varying degrees of thrombolytic activity within this range. The IC_50_ value for the leaf extract was measured as 275.06 *µ*g/mL. This IC_50_ value indicates that the leaf extract achieves a 50% reduction in clot formation at a concentration of 275.06 *µ*g/mL. The stem extract also displayed a range of clot lysis percentages, ranging from 31.8% to 49.6%. The IC_50_ value for the stem extract was 242.74 *µ*g/mL. This value indicates that the stem extract effectively reduces clot formation by 50% at a 242.74 *µ*g/mL concentration. The root extract demonstrated a range of clot lysis percentages from 9.6% to 39.8%. The IC_50_ value for the root extract was 319.62 *µ*g/mL. This indicates that the root extract achieves a 50% reduction in clot formation at a concentration of 319.62 *µ*g/mL. The thrombolytic activity may be due to alkaloids and tannins [36, 37]. Thus, the thrombolytic, anti-inflammatory, and antioxidant properties of *D. lindleyi* may be due to its alkaloids, phenolic compounds, and other phytocompounds [38]. Statistical analysis also indicates a significant positive correlation between the antioxidant, anti-inflammatory, and thrombolytic activities with the content of alkaloids and tannins [39, 40].

## 5. Conclusion

In summary, the results indicate that the different parts of *D. lindleyi* (root, stem, and leaf) have varying levels of effectiveness in their antioxidant, thrombolytic, and anti-inflammatory activities. The root extract is the most effective as an antioxidant, the stem extract has the highest thrombolytic activity, and the leaf extract is the most potent in anti-inflammatory activity. These findings can have implications for potential applications of these plant extracts in health and medicine. However, further research is usually necessary to fully understand their mechanisms of action and potential benefits.

### 5.1. Limitations of the Study

The research on *D. lindleyi*, a high-altitude plant with no recorded medicinal use, aimed at examining its phytochemical properties and evaluating its potential as an antioxidant, anti-inflammatory, and thrombolytic agent during the COVID-19 pandemic. However, the study was limited by the need for in vivo testing, which could not be conducted due to time constraints.

## Figures and Tables

**Table 1:** Qualitative test for alkaloids.

Plant parts used	Alkaloid detecting reagents				
	*D*	*T*	*W*	*H*	*M*
Leaf	++	++	+++	+++	+++
Stem	+++	+	++	+++	+
Root	++	_	++	+++	+++

*Note*. *D* = Dragendroff”s reagent, *H* = Hager's reagent, *M* = Mayer's reagent, *W* = Wagner's reagent, and *T* = tannic acid reagent.

**Table 2:** Qualitative test of twelve other phytochemicals.

Plant parts	Flv.	Sap.	Tan.	Phe.	Ter.	Str.	Gly.	C.Gly.	Qui.	Cou.	Pro.	Res.
Leaf	++	+	+++	++	+++	+	+	++	+++	+++	+++	—
Stem	+++	+	++	++	+++	++	—	+++	+++	+++	++	+
Root	++	—	+++	+++	+++	++	+	++	+++	++	+++	++

*Note.* Cou. = coumarin, C.gly. = cardiac glycosides, Flv. = flavonones, Gly. = glycosides, Phe. = phenol, Pro. = protein, Res. = resins, Sap. = saponins, Str. = steriods, Tan. = tannins, Ter. = terpenoids, and Qui. = quinine.

**Table 3:** Results of regression correlation analysis of the total content in bioassays.

Name	Protein	Flavonoid	Phenol	Tannin	Alkaloid
*R* ^2^	0.5273	0.2892	0.2606	0.9574	0.9808
*R*	0.7	0.54	0.51	0.9^*∗*^	0.96^*∗*^

*Note. R* = correlation coefficient, *R*^2^ = linear regression coefficient, and ^*∗*^ = significant.

## Data Availability

All data in this manuscript are available from the corresponding author upon request.
